# Guanylate cyclase C reduces invasion of intestinal epithelial cells by bacterial pathogens

**DOI:** 10.1038/s41598-018-19868-z

**Published:** 2018-01-24

**Authors:** Surya Amarachintha, Eleana Harmel-Laws, Kris A. Steinbrecher

**Affiliations:** 1 Division of Gastroenterology, Hepatology and Nutrition, Cincinnati Children’s Hospital Medical Center, Cincinnati, Ohio 45229 USA; 2 Department of Pediatrics, University of Cincinnati College of Medicine, Cincinnati, Ohio 45229 USA

## Abstract

The guanylate cyclase C (GC-C) receptor regulates electrolyte and water secretion into the gut following activation by the *E. coli* enterotoxin STa, or by weaker endogenous agonists guanylin and uroguanylin. Our previous work has demonstrated that GC-C plays an important role in controlling initial infection as well as carrying load of non-invasive bacterial pathogens in the gut. Here, we use *Salmonella enterica* serovar Typhimurium to determine whether GC-C signaling is important in host defense against pathogens that actively invade enterocytes. *In vitro* studies indicated that GC-C signaling significantly reduces *Salmonella* invasion into Caco2-BBE monolayers. Relative to controls, GC-C knockout mice develop severe systemic illness following oral *Salmonella* infection, characterized by disrupted intestinal mucus layer, elevated cytokines and organ CFUs, and reduced animal survival. In *Salmonella*-infected wildtype mice, oral gavage of GC-C agonist peptide reduced host/pathogen physical interaction and diminished bacterial translocation to mesenteric lymph nodes. These studies suggest that early life susceptibility to STa-secreting enterotoxigenic *E. coli* may be counter-balanced by a critical role of GC-C in protecting the mucosa from non-STa producing, invasive bacterial pathogens.

## Introduction

Infectious diarrheal disease is a significant cause of morbidity and mortality in the developing world^1^. Diarrheal disease, such as that caused by enterotoxigenic *Escherichia coli* (ETEC), kills approximately 500,000 children each year^2^. Deregulated fluid secretion during ETEC infection can be mediated in part by peptide enterotoxins such as heat stable (STa) peptide variants. STa is thought to bind a single lumen-oriented receptor in the intestine known as guanylate cyclase 2 C (Gucy2C, hereafter referred to as GC-C)^3–5^. GC-C and its endogenously produced ligands (guanylin (Gn) and uroguanylin (Ugn)) regulate cyclic guanosine monophosphate (cGMP) production in the epithelia of the intestine^6,7^. Release of Gn and Ugn into the gut lumen binds to GC-C, elevates intracellular cGMP, and activates protein kinase G II (PKGII)^8,9^. In many cell types, studies indicate that cGMP signaling may directly, or by regulating phosphodiesterase activity, cross-activate cyclic adenosine monophosphate (cAMP) signaling pathways as well^10–12^. A critical endpoint of GC-C signaling is secretion of chloride and bicarbonate ions by cystic fibrosis transmembrane conductance regulator (CFTR) and loss of sodium import by Na+/H+ Exchanger 3 (NHE3). The resulting accumulation of extracellular electrolytes pulls water into the gut lumen and has an important hydrating effect on the luminal contents. ETEC STa has a super-agonist effect on GC-C, causing copious electrolyte and water secretion. Importantly, unlike STa, physiological activation of GC-C by Gn or Ugn is well controlled and does not cause secretory diarrhea.

Proper hydration of the intestine by coordinated CFTR and NHE3 activity is essential for homeostatic balance between host and microflora. In addition to persistent lung pathology, patients with CFTR mutation have significant intestinal disease characterized by viscous mucoid obstructions, bacterial overgrowth, mucosal immune activation, and inflammation^13^. CFTR is necessary for mucin hydration and an effective physical barrier between the epithelial monolayer and luminal bacteria. In CFTR deficiency, a chronic close physical association between microflora and host epithelia induces mucosal inflammation^13,14^. Similarly, poor sodium absorption due to mutation of human NHE3 causes congenital diarrhea syndrome and intestinal inflammation and deletion of the exchanger in mice leads to pro-inflammatory gene expression, enhanced bacterial adhesion and translocation, and colitis^15–17^.

Several lines of evidence indicate that GC-C signaling, as an upstream cascade leading to CFTR and NHE3, is also essential for intestinal fluid homeostasis. Human kindred having congenital GC-C mutations reveal that it has an important role in linking luminal hydration to infection and inflammation^18,19^. GC-C loss-of-function mutations cause meconium ileus in infants and these individuals retain an increased susceptibility to bowel infection as they age^18^. Interestingly, gain-of-function mutations in GC-C also cause significant intestinal pathology including secretory diarrhea, alterations in gut microbiota, and susceptibility to inflammatory bowel disease^19–21^. Using several model systems, our previous work demonstrates that GC-C signaling plays a crucial role in host defense during inflammation and infection^22–24^. Specifically, GC-C is essential during infection with non-invasive attaching/effacing (AE) lesion-forming bacterial pathogens in order to reduce bacterial load and minimize systemic dissemination^25^. However, little is understood about a broader role for GC-C during bacterial infection. Specifically, a detailed understanding of the impact of electrolyte and fluid regulation by GC-C and differing mechanisms of host-bacteria interactions is lacking. For example, it remains unclear whether cGMP-dependent secretion affects adherence and invasion by more aggressive, cell-penetrating bacteria. Accordingly, we hypothesize that activation of the GC-C signaling pathway and its associated secretory response reduces pathology and systemic spread of enteric invasive bacterial species. In this study, we turn to an invasive bacterial pathogen, *Salmonella enterica* serovar Typhimurium (hereafter referred to as *Salmonella*), to investigate a role for GC-C in directly regulating host-pathogen interactions. We demonstrate using cell culture and mouse model systems that GC-C signaling in epithelia of the gut is critical for minimizing bacterial invasion into enterocytes and that it is essential for animal survival during infection by invasive bacterial pathogens.

## Results

### Cells lacking GC-C are prone to *Salmonella* invasion

**Figure 1:**
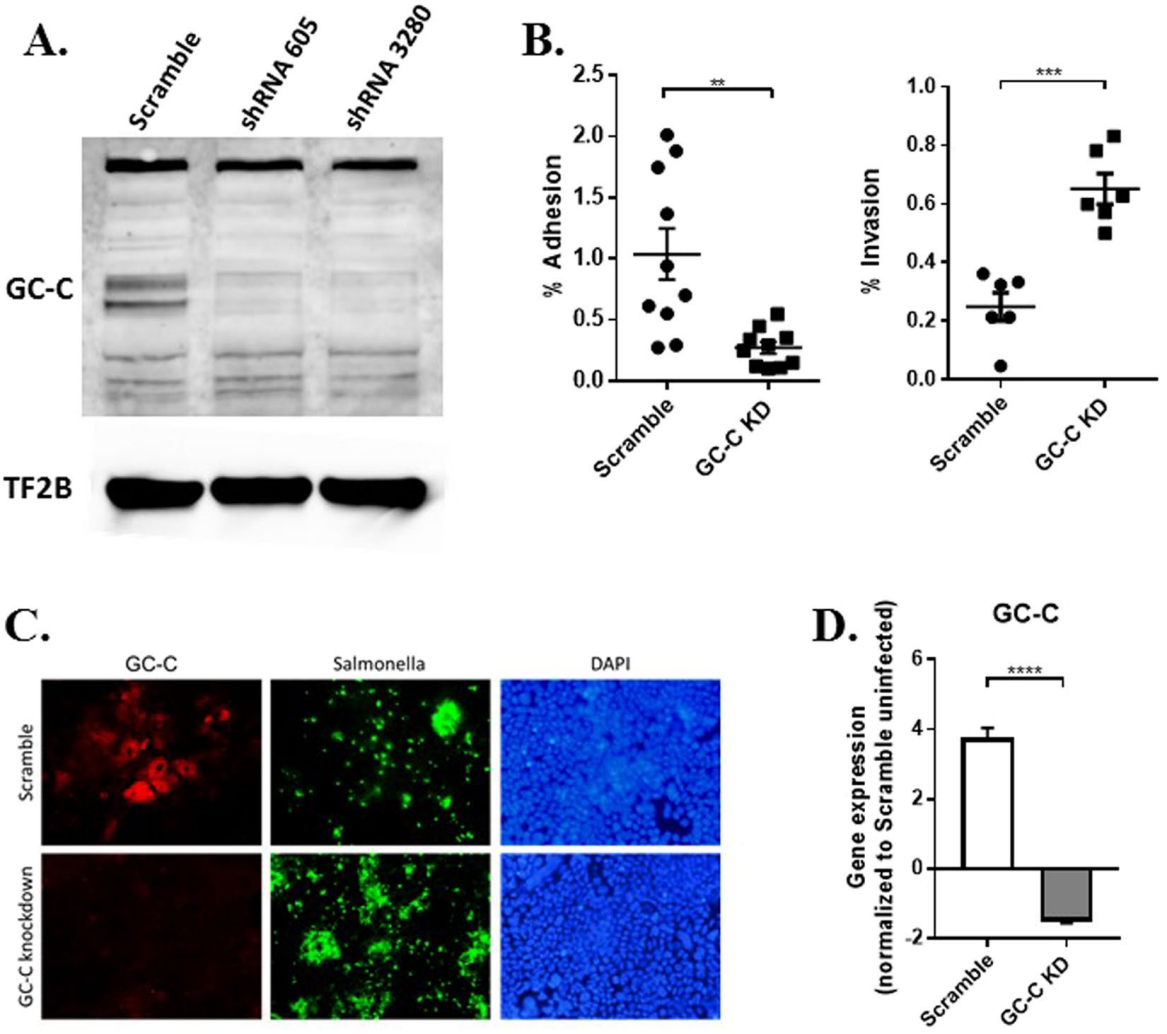
Elevated *Salmonella* invasion in GC-C knockdown cells. (**A**) Immunoblot confirming GC-C knockdown using two lentivirus-based shRNAs (shRNA 605 or shRNA 3280). An shRNA of scrambled sequence was used as control and general transcription factor IIB (TF2B) was used to confirm even loading. (**B**) Adhesion assays and gentamicin protection invasion assays were performed in confluent shRNA 3280 GC-C knockdown (GC-C KD) and control (scramble) cells. Experiments were conducted in triplicate using three different cell line clones. (**C**) Immunofluorescence of Caco2-BBE cells showing GC-C expression and invasion of *Salmonella* in GC-C knockdown cells. Confluent cells were infected for 90 minutes, treated with gentamicin, fixed and stained with GC-C (TRITC) or *Salmonella* (FITC) antibodies. 4′,6-diamidino-2-phenylindole (DAPI) was used to stain the cell nuclei. Images were taken at 200x. (**D**) Scramble and GC-C KD cells were infected with *Salmonella* for four hours and then lysed to collect RNA. Realtime RT-PCR was performed on GC-C. n = 8. **P < 0.01, ***P < 0.001, ****P < 0.0001.

We began by using a well-described *in vitro* cell culture system to investigate epithelial cell-bacteria interactions. Caco2-BBE cell monolayers, which express GC-C and downstream signaling components, were used to determine the impact of GC-C deletion on *Salmonella* adherence and invasion. We chose this pathogen for these studies because it provides an effective, well-characterized research model of *in vitro* and *in vivo* bacterial infection. This approach allows for direct analysis of bacterial adhesion and invasion of enterocytes and contrasts nicely with our previous work using an AE lesion-forming species. In addition, *Salmonella* infection is of high clinical relevance, with millions of cases of enteric disease and systemic salmonellosis occurring each year^26,27^. We transduced cells with virus expressing a scrambled shRNA sequence as control or one of two separate GC-C targeting sites (shRNA605 and shRNA3280). Western blot confirmed efficient GC-C knockdown in puromycin selected cells and further studies were performed with scramble control and GC-C shRNA 3280 (Fig. 1A). Upon *Salmonella* infection (using a multiplicity of infection (MOI) of 100) for 30 minutes, we measured lower adhesion of bacteria to GC-C knockdown cells (Fig. 1B). Conversely, GC-C knockdown led to elevated numbers of *Salmonella* that had invaded the cell monolayer after 90 minutes (Fig. 1B). This was confirmed by fluorescence microscopy showing significant *Salmonella* uptake in GC-C knockdown Caco2-BBE cells (Fig. 1C). Expression of GC-C mRNA was measured four hours after bacterial infection and found to be elevated relative to uninfected control cells and still diminished in shRNA-expressing cells (Fig. 1D). These results suggest that GC-C signaling plays an important role in regulating host-bacteria interactions as well as bacterial invasion into enterocytes.

### Activation of GC-C by peptide agonists minimizes *Salmonella* invasion

**Figure 2:**
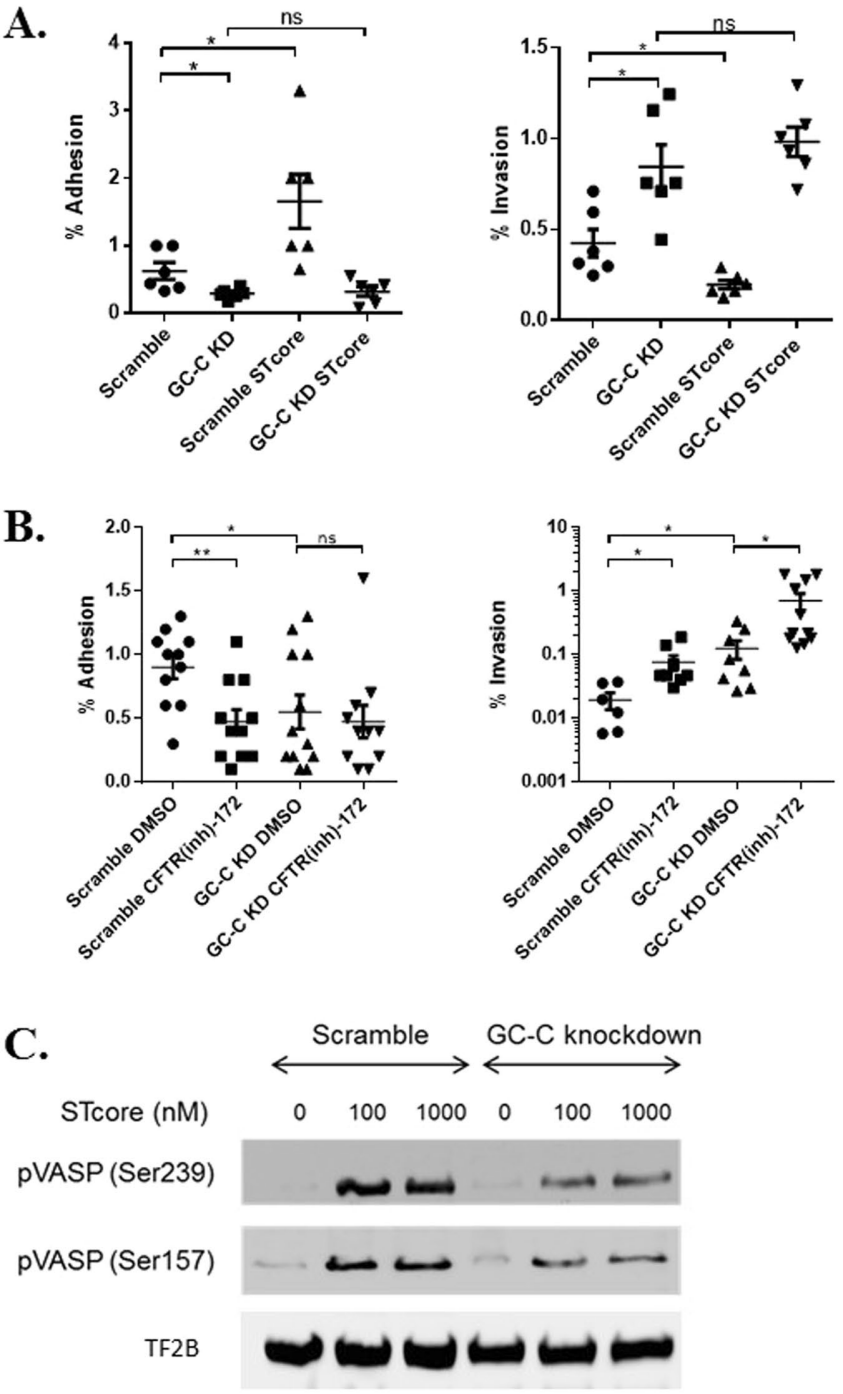
Activation of GC-C by STcore reduces *Salmonella* invasion. (**A**,**B**) Adhesion and invasion assays in scramble and GC-C knockdown (GC-C KD) cells pretreated with STcore or CFTR inhibitor (CFTR(inh)-172). (**C**) Immunoblot of protein lysates from scramble and GC-C knockdown cell lines treated without or with STcore for 30 minutes. VASP phosphorylation (pVASP) at sites Ser239, Ser157 was measured while general transcription factor IIB (TF2B) was used as loading control. *P < 0.05, **P < 0.01, ns: not significant.

We reasoned that supplementing the existing low level of GC-C activation would serve to further minimize *Salmonella* invasion. We used STcore which is a GC-C-binding 14 amino acid derivative of ETEC STa that is nearly identical to linaclotide, a GC-C agonist approved by the FDA for treatment of irritable bowel syndrome^28^. Scramble and GC-C knockdown Caco2-BBE cells were pretreated with STcore 15 minutes prior to *Salmonella* infection and then host-cell interaction assays were performed. These studies revealed an interesting inverse correlation between adhesion and invasion following STcore-activation of GC-C. While adhesion increased with STcore treatment in scramble shRNA control cells, invasion was substantially decreased (Fig. 2A). As expected due to the absence of GC-C, no such differences were noted in knockdown cells. Because CFTR is an important downstream target of GC-C/cGMP signaling, we next blocked CFTR in control and GC-C knockdown cells and measured *Salmonella* adhesion and invasion. We found that blocking CFTR mimicked loss of GC-C with regard to *Salmonella* attachment and cell entry (Fig. 2B). Further, while loss of both GC-C and CFTR activity did not change numbers of bacteria adhered to the cell layer (Fig. 2B, left), there was a notable and statistically significant increase in bacterial invasion in GC-C/CFTR-inhibited cells relative to all other treatment groups (Fig. 2B, right). To confirm strong activation of GC-C receptor signaling pathways, we performed immunoblot for phosphorylated vasodilator-stimulated phosphoprotein (VASP) at phosphorylation sites Ser239 and Ser157 (Fig. 2C). Phosphorylation of VASP in control cells was evident upon treatment with STcore and to a much lesser degree in GC-C knockdown cells, confirming that GC-C activation by exogenous peptides leads to cGMP signaling and crosstalk to cAMP-dependent pathways. Collectively, these results suggest that activation of the GC-C pathway plays a potent defensive role in preventing movement of invasive pathogens like *Salmonella* into intestinal epithelial cells.

### GC-C is essential for maintaining mucus layer integrity and minimizing systemic spread of *Salmonella* in mice

**Figure 3:**
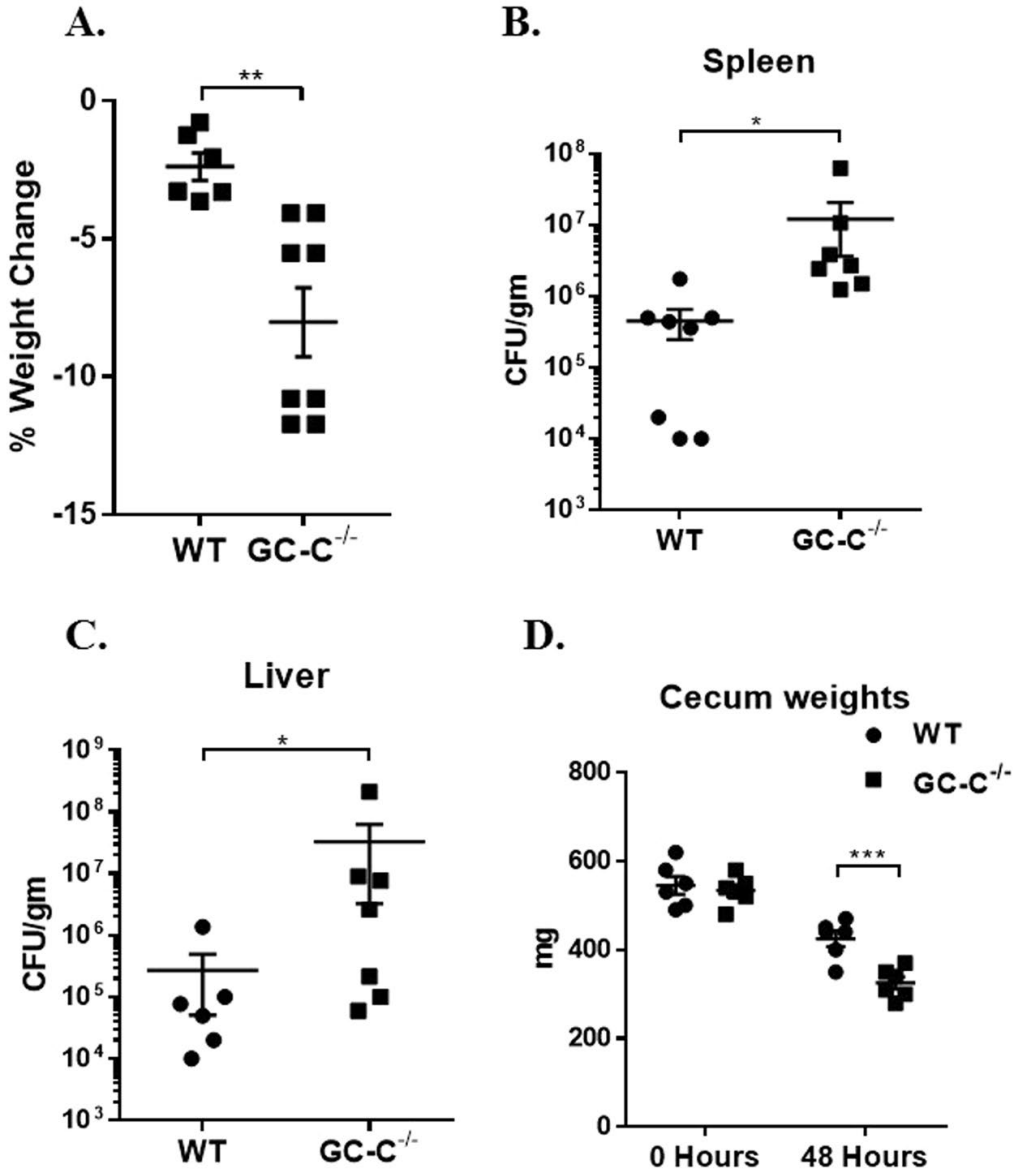
GC-C knockout mice show significant weight loss and increased systemic dissemination upon oral *Salmonella* infection. Mice were infected by oral gavage with 10^7^ *Salmonella*. (**A**) Weight loss was calculated based on the initial weights. (**B**,**C**) *Salmonella* CFUs were measured after two days of infection in spleen and liver. (**D**) Ceca were collected and weighed from uninfected and infected mice (48 hrs). *P < 0.05, **P < 0.01, ***P < 0.001.

In an effort to better understand the importance of GC-C-regulated cGMP production on host-bacteria interactions, we next used an *in vivo* murine model of *Salmonella* infection that is similar to human typhoid fever. We chose this approach because it allows us to investigate systemic dissemination of an enteric pathogen without significant epithelial architecture distortion or ulceration at early time points. GC-C knockout mice were infected with 10^7^ colony forming units (CFU) *Salmonella* using oral gavage. Significant weight loss was observed in knockout mice one day post-infection (Fig. 3A). Spleen and liver (Fig. 3B,C) were collected at study day 2 and showed significant increases in *Salmonella* CFUs in GC-C−/− mice as compared to WT. There was a significant loss in ceca weight in infected knockout mice compared to control group (Fig. 3D).

**Figure 4:**
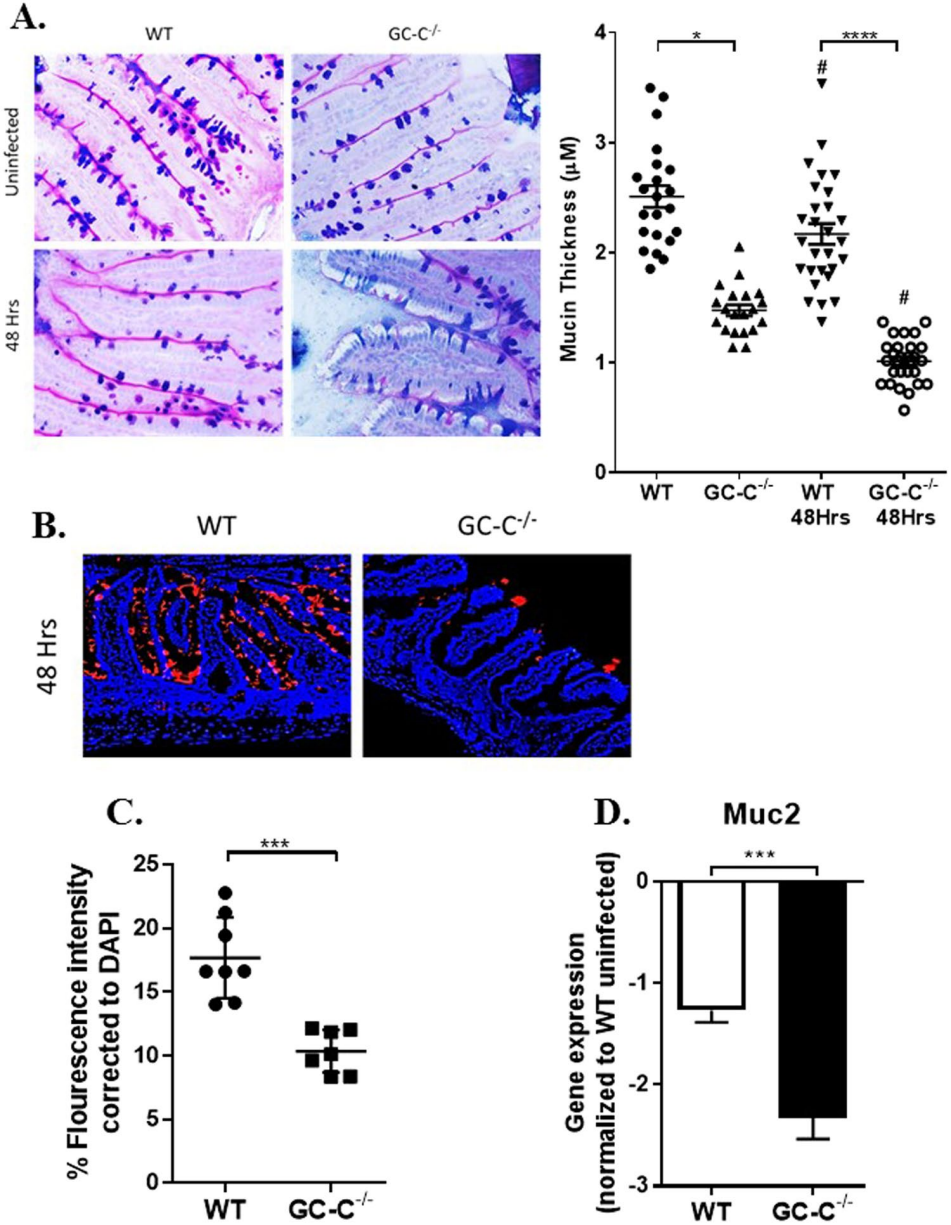
Mice lacking GC-C produce significantly less mucin at baseline and two days after *Salmonella* infection. (**A**) Ilea from naïve mice and those infected (10^7^) with *Salmonella* for 48 hrs were stained with alcian blue and PAS. Thickness of the adherent mucus layer in both naïve and infected mice was measured using cellSens software. Images were obtained at 400X. (n = 6 mice and 2–5 images per mouse). (**B**) Ilea of infected mice were stained for mucin 2 (red) and cell nucleus was visualized using DAPI. Images were obtained at 200X. (**C**) Muc2 fluorescence was quantified and then normalized with DAPI intensity. (**D**) Realtime RT-PCR analysis of GC-C−/− ileum at 48 hrs post-infection showed a clear decrease in mucin 2 (Muc2) levels, n = 6–8 mice. *P < 0.05, ***P < 0.001, ****P < 0.0001, ^#^P < 0.05 relative to naïve mice of same genotype.

GC-C signaling is a potent regulator of chloride, bicarbonate, and water secretion and therefore has a putative role in production and/or expansion of intestinal mucus. We next determined if poor pathogen containment could be attributed, in part, to defects in the intestinal mucus layer in GC-C knockout mice. We focused on terminal ileum in these studies because small bowel is an important entry point in this model of *Salmonella* infection. Ileum from naïve and infected mice were processed through alcian blue and periodic acid-Schiff (PAS) stains to visualize mucins (Fig. 4A). Adherent, closely associated mucus was clearly evident as a thin pink line on the outer epithelial cell membrane. Thinning was more severe upon *Salmonella* infection in GC-C−/− animals and in some portions of the GC-C−/− bowel the mucus layer was completely lost. Similar results were noted in GC-C−/− colon. Measurements of mid-villus mucus thickness confirmed a significantly reduced layer in the knockout mice compared to WT in both naïve and infected states (Fig. 4A, right). Immunofluorescence and quantification of mucin 2 (Muc2), the primary secreted mucin in the intestine demonstrated a significant loss in *Salmonella*-infected GC-C knockout mice (Fig. 4B,C). Further, realtime RT-PCR indicated that infection significantly reduced Muc2 mRNA in the ileum of GC-C−/− mice relative to WT animals (Fig. 4D).

**Figure 5:**
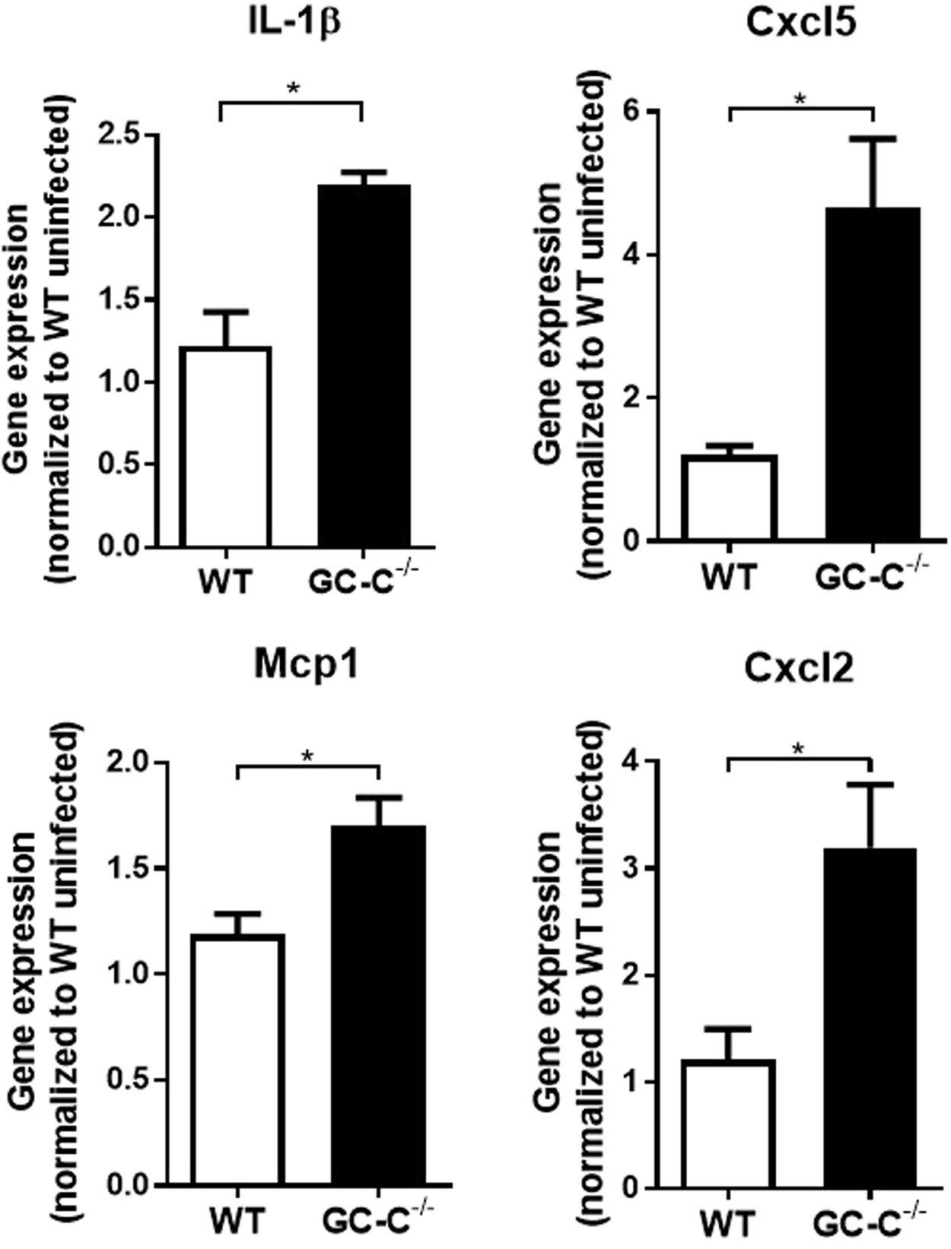
Cytokine and chemokine expression is elevated in *Salmonella*-infected GC-C knockout mice relative to control. Total RNA was extracted from ileum tissue of *Salmonella* infected mice at day 2. Cytokine and chemokine expression was performed using realtime RT-PCR and demonstrated an increase in interleukin 1 beta (IL-1β), monocyte chemoattractant protein 1 (Mcp-1), C-X-C motif chemokine ligand 2 (Cxcl2), and C-X-C motif chemokine ligand 5 (Cxcl5) in GC-C−/− mice. Gene expression was normalized to total RNA collected from ileum of uninfected WT control mice. n = 6–8mice. *P < 0.05.

Poor mucus layer homeostasis coupled with *Salmonella* translocation predicted pro-inflammatory gene expression in the intestine of GC-C−/− mice. Relative to naïve wildtype controls, realtime RT-PCR demonstrated elevated cytokine and chemokine expression in all mice on infection day 2. Notably, there was an increase in interleukin 1 beta (Il1b), monocyte chemoattractant protein 1 (Mcp1), C-X-C motif chemokine ligand 2 (Cxcl2), and C-X-C motif chemokine ligand 5 (Cxcl5) in infected knockout mice compared to WT infected (Fig. 5). Collectively, this series of studies suggest that the GC-C signaling pathway plays an important role in establishing or maintaining the intestinal mucus layer and in minimizing bacterial pathogen invasion and inflammatory gene expression in the intestine.

### Infection with high levels of *Salmonella* leads to poor survival of GC-C knockout mice

**Figure 6:**
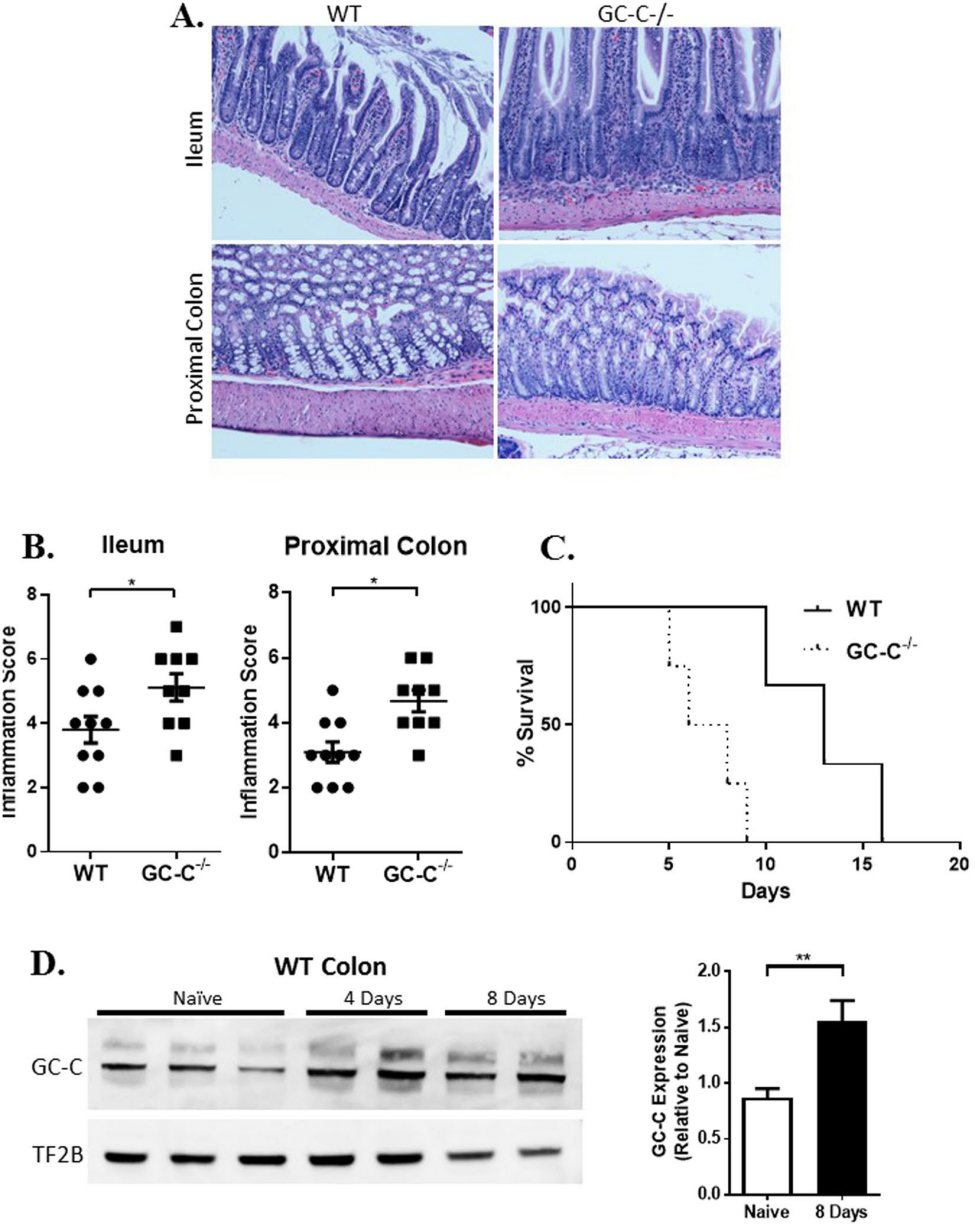
*Salmonella* infection causes intestinal inflammation and decreased survival in GC-C knockout mice. Mice were dosed with 10^9^ CFU *Salmonella*. (**A**) H&E staining of ileum and proximal colon from day 4 infected mice revealed more severe inflammation in GC-C−/− mice. Images were obtained at 200X. (**B**) Histopathology scoring of inflammation in ileum and proximal colon. **(C)** Decreased survival in mice lacking GC-C. (**D**) At days 4 and 8, whole colon protein extracts from wildtype mice were immunoblotted for GC-C and compared to naïve. Day 8 bands from multiple gels were quantitated and graphed. n = 10–12mice. *P < 0.05, **P < 0.01.

To investigate the role of GC-C signaling during a more robust infection and over a longer time frame, we increased the *Salmonella* dose to 10^9^ CFU and analysis time points to 4 days and beyond. Significant differences in weight loss between WT and GC-C−/− mice were noted at day four (WT 6.55% ± 2.001, GC-C−/− 12.65% ± 1.177, P < 0.05). An elevated initial dose of *Salmonella* and a longer experimental time point prompted us to assess mucosal inflammation in terminal ileum and proximal colon tissues. Although inflammation was mild to moderate, there was increased mixed immune cell infiltrate in both GC-C knockout mice as compared to WT (Fig. 6A,B). In some studies, we allowed the infection to progress past 4 days and found the GC-C knockout animals developed significant morbidity. Survival of GC-C−/− mice began to drop off as early as day 5 and none of the knockout mice lived past day 10 (Fig. 6C). WT animals survived significantly longer, often past day 15 (P < 0.05). Importantly, western blotting revealed elevated levels of GC-C protein in wildtype animals during days 4 and 8 of the infection (quantitation on day 8 showed an approximately 50% increase; Fig. 6D).

### Disease course during intravenous *Salmonella* infection is not affected by GC-C deletion

While the intestine is the predominant site of GC-C expression, this receptor can be found outside the gut. To understand if extra-intestinal GC-C signaling affects the course of *Salmonella* infection once it translocates from the gut, mice were infected intravenously with *Salmonella*. We found no genotype-dependent differences in weight loss or liver and spleen CFUs (Supp. Fig. S1). These results suggest that that extra-intestinal expression of GC-C does not play a role in systemic *Salmonella* spread or expansion.

### Exogenous GC-C agonist peptide reduces host-pathogen interactions and bacterial translocation

**Figure 7:**
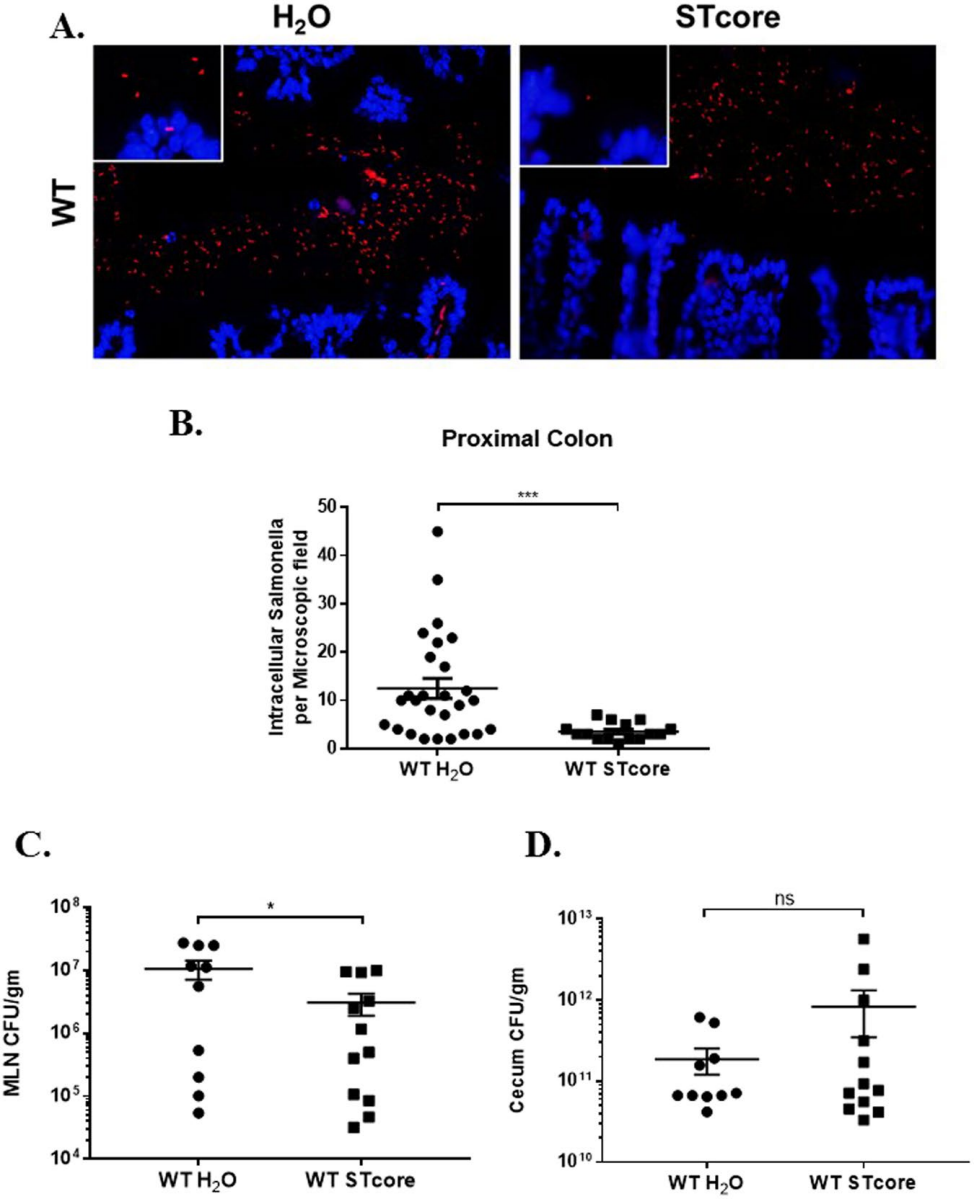
Activation of GC-C with an exogenous peptide minimizes host-pathogen interactions. Antibiotic-pretreated wildtype mice were infected with *Salmonella* (10^7^), treated with repeated doses of STcore, and analyzed 24 hours later. (**A**) Fluorescent *in situ* hybridization was used to visualize *Salmonella* in colon of wildtype water- or STcore-treated mice. (400X, insets 1000X). (**B**) Numbers of *Salmonella* that had entered or moved below the epithelial cell layer were counted. N = 6–8 mice per group with 2–3 fields per mouse ***P < 0.001. (**C**) Mesenteric lymph nodes (MLN) were homogenized and plated in order to determine *Salmonella* colony forming units. *P < 0.05 n = 10–12 mice. (**D**) *Salmonella* load was determined in whole, unflushed cecum. n = 10–12 mice. ns: not significant.

We next determined if therapeutic activation of GC-C in wildtype animals could reduce bacterial invasion of intestinal epithelia and translocation from the gut. We again used the GC-C agonist STcore because it can be orally delivered and stimulates GC-C throughout the length of the intestinal tract. We focused on localization of *Salmonella* within the intestine as well as bacterial load in mesenteric lymph nodes as an indicator of *Salmonella* translocation. Fluorescent *in situ* hybridization analysis showed *Salmonella* closely adhered and penetrating the epithelial cell layer of control wildtype mice that had been treated with water (Fig. 7A, left). Wildtype mice dosed with STcore, however, clearly indicated that robust GC-C activation reduced host-pathogen co-localization as *Salmonella* adherence and movement into the epithelia were substantially minimized (Fig. 7, right). Using high power microscopy, we quantitated the number of *Salmonella* that had invaded into or completely penetrated the epithelial cell layer and found this to be significantly reduced in the context of exogenous peptide activation of GC-C (Fig. 7B). Consistent with these data, we found that *Salmonella* bacterial load in mesenteric lymph nodes of STcore-treated wildtype mice was significantly decreased as compared to control animals (Fig. 7C). Notably, GC-C agonist did not impact overall levels of *Salmonella* colonization in the intestine as bacterial numbers in whole, unflushed cecum (cecum tissue and luminal contents) were similar in water and STcore-treated mice (Fig. 7D).

## Discussion

The current study suggests an important role for GC-C and its ligands in protecting the intestine from invasive bacterial pathogens. To our knowledge, this is the first study showing that GC-C-dependent signaling pathways directly affect physical interactions between invasive bacterial pathogens and the epithelial cell layer of the gut. Using a well-controlled *in vitro* model system, we show that signaling from the GC-C receptor controls bacterial adherence and invasion into epithelial cells. Enhanced invasion through the epithelial layer and subsequent pathogen dissemination is likely central to the rapid death of GC-C null mice following *Salmonella* infection. These data expand our understanding of the importance of GC-C during infancy. One aspect of childhood susceptibility to ETEC is elevated GC-C expression during the first few years of life^29^. We suggest that this early life susceptibility to STa-secreting ETEC is counter balanced by the critical role of GC-C in protecting the mucosa from non-STa producing, invasive bacterial pathogens, such as *Salmonella*. Concomitant with robust GC-C activity in the infant intestine is the colonization and expansion of commensal microflora and we suggest that additional studies will be necessary to determine if GC-C-regulated microflora complexity impacts susceptibility to invasive pathogen infection^20,25,30^.

Production, secretion, and extracellular expansion of intestinal mucins are an essential aspect of intestinal innate immunity^31^. Intestinal mucins are secreted as granulae by goblet cells and expand in the gut lumen. Critical to this process is chloride and bicarbonate secretion by CFTR^32,33^. Expression of CFTR, GC-C, and Ugn/Gn in enterocytes and/or mucin-secreting goblet cells provides a local environment that is rich in bicarbonate, chloride, and fluid^34^. There is a significant reduction of mucus release when bicarbonate and fluid transport are inhibited, suggesting the need for continuous secretion in order to maintain physiological mucus production and expansion^33^. This connection between electrolyte/water release and mucus hydration has important implications for susceptibility to bacterial pathogen infection. It was notable that we found fewer Muc2-positive goblet cells in *Salmonella*-infected GC-C knockout animals, raising the possibility that goblet cell ablation or dysfunction is an important cause of defective mucus layer in these mice. Importantly, poor mucus layer production and expansion in GC-C knockout mice may be related, in part, to elevated susceptibility to *Salmonella* infection.

It is likely that the defects in mucus layer deposition in mice lacking GC-C are only partially responsible for high levels of bacterial invasion during *Salmonella* infection. Using an *in vitro* model system that produces a negligible mucus layer, we observed diminished adherence but elevated invasion of *Salmonella* in GC-C knockdown cells as compared to control. We further demonstrated that activation of GC-C above baseline using exogenous ligand (STcore peptide) yields fewer numbers of intracellular *Salmonella*. While we found STcore to be an important experimental reagent in these studies, significant uncertainty remains about any therapeutic use of GC-C agonists in the context on on-going Salmonella infection. A likely mechanism for reduced bacteria internalization into epithelial cells is GC-C-dependent chloride and water secretion at the epithelial surface. Work by others has shown that *Salmonella* internalize and translocate at diminished levels in the context of chloride and water secretion elicited by a variety of secretagogues^35^. We demonstrate that blockade of CFTR also enhances bacterial invasion and that this elevates cell entry caused by GC-C knockdown alone. While highly suggestive, additional work will be needed to determine if this indicates that GC-C blocks bacterial invasion through both CFTR-dependent and –independent pathways. Relevant to this notion is the increased phosphorylation of VASP in cells treated with STcore, supporting a putative role for GC-C signaling in actin cytoskeleton reorganization and the mechanics of *Salmonella* uptake^36^. Phosphorylation of VASP at Ser239 and Ser157 is mediated by PKGII and Protein Kinase A, respectively^37^. This supports previous suggestions of a role for GC-C in direct activation of cGMP-dependent kinases as well as crosstalk with cAMP signaling pathways through cGMP-regulated phosphodiesterases^10,11,38^. Collectively, these data underscore the importance of this signaling pathway to enteric bacterial pathogenesis and identify areas needing further experimental work to fully determine the mechanistic role for this receptor in pathogen adhesion and invasion in the intestine.

Taken together, our work suggests that GC-C signaling plays a critical role in preventing uptake of invasive bacterial pathogens and we provide *in vivo* proof-of-concept that activation of this pathway using orally available GC-C agonist peptides is protective during *Salmonella* infection (Supp. Fig. S2). Although therapeutic GC-C agonists have been extensively studied in the treatment of irritable bowel syndrome and chronic constipation, additional studies focusing on the impact of GC-C agonists on the bacterial microflora of the bowel, pathogenic or otherwise, is warranted^20^.

## Materials and Methods

### Mice

Animal procedures were performed according to guidelines approved by the Institutional Animal Care and Use Committee at Cincinnati Children’s. Wildtype and GC-C−/− mice used in this study were bred and housed in microisolator cages within the same room of the Cincinnati Children’s specific pathogen-free barrier facility^39–41^. Colony and study animals, both wildtype control and GC-C−/− mice, were genotyped according to established PCR-based protocols. Mice in all study groups were age and sex-matched and had been bred into a C57BL/6 J background for >10 generations.

### *Salmonella* Typhimurium infection

*Salmonella enterica* serovar Typhimurium type SL1344 was cultured as described elsewhere^42^. Most studies herein focused on systemic typhoid fever-like illness that allowed us to investigate bacterial translocation from non-inflamed intestine during the initial days of infection^43^. In some studies, we fasted mice for four hours and then gave 20 mg oral streptomycin one day prior to infection in order to facilitate greater gut pathogen load during fluorescence *in situ* hybridization imaging studies^44^. Bacterial culture concentration was estimated using optical density and confirmed by plating on the day of infection and counting colony forming units (CFUs). For all *Salmonella* infections, food was withdrawn for 4 hours and then mice were dosed by oral gavage (10^7^ or 10^9^ CFUs in 200 ul per mouse). Organ CFU analysis and inflammation scoring were performed as previously reported^22,25^. In some studies, wildtype mice were given 10 µg STcore, a 14 amino acid peptide based on *ETEC* STa, by oral gavage (−4 hrs, −1 hr, at infection, and +1 hr). For intravenous infection, mice were injected (10^4^ CFUs in 100 µl) via tail vein and analyzed 48 hours later.

### GC-C shRNA knockdown and adhesion/invasion assays

Lentivirus was produced in human 293T HEK cells using second generation lentivirus vectors and was transduced into the human intestinal Caco-2 Brush Border Epithelial (Caco2-BBE) cell line^45,46^. Caco2-BBE cells differentiate rapidly at confluence into a polarized monolayer with a well-developed brush border that expresses transporters and digestive enzymes found in small bowel^47–49^. We used two lentivirus short hairpin RNA (shRNA) constructs to target GC-C at different sites on the sequence (Cat#SHCLNG-NM_004963; GUCY2C MISSION^®^ shRNA Bacterial Glycerol Stock, Sigma) (Supp. Table 1). Lentivirus expressing a control, scrambled sequence was designed and is referred to as ‘scramble’. Caco2-BBEs were transduced and selected with puromycin in order to ensure uniform expression of shRNA^50^. Control (scramble) and GC-C knockdown (shRNA 3280) Caco2-BBE cells were grown at least one week past confluence. Assays were performed as previously described by Gagnon *et al*. with minor alterations^50^. After 24 hours of antibiotic-free media, *Salmonella* was added at 100MOI and incubated at 37 C in a humidified aerobic incubator for 30 min for each adhesion assay. Adhesion was expressed as the percentage of the CFUs from adhered bacteria to CFUs from total bacteria added to each well. For invasion assays, cells were incubated at 37 C for 90 minutes with 100MOI *Salmonella* after which media was removed. New media supplemented with 150 µg/mL gentamicin (cat# G1397, Sigma) was then added for 60 minutes to kill bacteria that had not been taken into the cell layer. Invasion efficiency was expressed as the percentage of colonies from the cells before (cell-associated) and after (cell-invaded) gentamicin treatment. In the studies using CFTR inhibitor and GC-C agonist, cells were treated with 10 μM CFTR(inh)-172 (Sigma; cat#C2992) and with 1 µM STcore at 90 minutes and 15 minutes, respectively, prior to addition of *Salmonella* to the media.

### Immunoblotting and immunofluorescence

Frozen ileum tissue or Caco2-BBE cells were homogenized and lysed in cold RIPA buffer to collect protein supernatant, immunoblots were performed, and band intensity quantitated as described previously^22,25^. Primary antibodies pVASP S239 (cat#3114) and pVASP S157 (cat#3111) were purchased from Cell Signaling. GC-C antisera has been published and validated previously^40^. Muc2 (SC-15334) antibody was used for immunofluorescence was performed as previously detailed (Santa Cruz Biotechnology)^25^. All images were taken with the same exposure times using an Olympus BX51 microscope running cellSens software (Olympus).

### Mucus measurements, fluorescence ***in situ*** hybridization, realtime RT-PCR

Tissues were extracted so as not to disturb luminal contents and immediately fixed in Carnoy’s solution or frozen in OCT compound as previously described^25^. Mucus measurements were obtained following alcian blue and periodic acid-Schiff staining using calibrated imaging software (cellSens, Olympus). Tissue sections were incubated with 1 μM *Salmonella* FISH DNA Oligo (5′-/5TexRd-XN/TCT CTG GAT TCT GTG GA-3′) at 55 °C for 90 min and at 37 °C overnight. Tissues were then washed and counter-stained with DAPI to visualize nuclei. All images were taken with the same exposure times using an Olympus BX51 microscope running cellSens software (Olympus). Realtime RT-PCR was performed as previously described and primer sequences are listed in Supplemental Table S1^51^.

### Statistical analysis

Data were analyzed using t test or ANOVA using GraphPad Prism software. For normally distributed data, significance was determined using an unpaired t test. For data sets found not to be distributed normally, we used nonparametric Mann-Whitney t test. Analysis of variation was coupled with Tukey’s post-hoc test. Data in figures are expressed as mean ± SEM and P values of <0.05 were considered to be statistically significant. All data presented herein was generated from three or more independent experiments.

## Electronic supplementary material

Supplementary Information
