# Supplementary material for: Guanylate cyclase C reduces invasion of intestinal epithelial cells by bacterial pathogens
Source: Sci Rep. 2018 Jan 24;8:1521. doi: 10.1038/s41598-018-19868-z (PMC5784150; doi:10.1038/s41598-018-19868-z)

Guanylate cyclase C reduces invasion of intestinal epithelial cells by bacterial pathogens

Surya Amarachintha[1], Eleana Harmel-Laws[1], Kris A. Steinbrecher[1, 2,*]

[1]Division of Gastroenterology, Hepatology and Nutrition, Cincinnati Children's Hospital Medical Center, Cincinnati, Ohio 45229, [2]Department of Pediatrics, University of Cincinnati College of Medicine, Cincinnati, Ohio 45229.

Supplemental Data:

Supplemental Table S1: Mouse and Human Realtime RT-PCR Primers and Human shRNA sequences

Supplemental Figure S1. Intravenous infection of *Salmonella* has no significant effect on weight loss and tissue bacterial load across genotypes

Supplemental Figure S2. GC-C-mediated host defense during *Salmonella* infection.

Immunoblot Images

Supplemental Table S1: Mouse and Human Realtime RT-PCR Primers and Human shRNA sequences

**Mouse Gene**	**Forward Primer**	**Reverse Primer**
Muc2	5’GTCTGCCACCTCATCATGGA3’	5’CAGGCAAGCTTCATAGTAGTGCTT3’
Il1b	5’AAGGGCTGCTTCCAAACCTTTGAC3’	5’ATACTGCCTGCCTGAAGCTCTTGT3’
Mcp1	5’CTTCTGGGCCTGCTGTTCA3’	5’CCAGCCTACTCATTGGGATCA3’
Mip2	5’GAGCTTGAGTGTGACGCCCCCAGG3’	5’GTTAGCCTTGCCTTTGTTCAGTATC3’
Cxcl5	5’AGCTGCGTTGTGTTTGCTTAACCG3’	5’TTGAACACTGGCCGTTCTTTCCAC3’
Rpl19	5’ATGAGTATGCTCAGGCTACAGA3’	5’GCATTGGCGATTTCATTGGTC3’
**Human Gene**	**Forward Primer**	**Reverse Primer**
GC-C	5’TATGTGTGGTGGTCCAGAGTTCCT3’	5’AATTCCCAGGAGACAGCGTCAGAA3’
RPL13	5’CCTGGAGGAGAAGAGGAAAGAGA3’	5’TTGAGGACCTCTGTGTATTTGTCAA3’
**shRNA**	**Targeting Sequence**	
sh605	5’CCGGCCTGGAGCACTTCGTATGTTTCTCGAGAAACATACGAAGTGCTCCAGGTTTTT3’	
sh3280	5’CCGGAGGTATAAGGACTCACACAAACTCGAGTTTGTGTGAGTCCTTATACCTTTTTT3’	

Supplemental Figure S1. Intravenous infection of *Salmonella* has no significant effect on weight loss and tissue bacterial load across genotypes. A. WT and knockout mice were intravenously injected with 10^4 CFUs Salmonella per mouse. Weight loss was calculated relative to initial body weights. **B., C.** Spleen and liver tissue were collected at 48 hours post-infection, plated, and CFUs were counted.

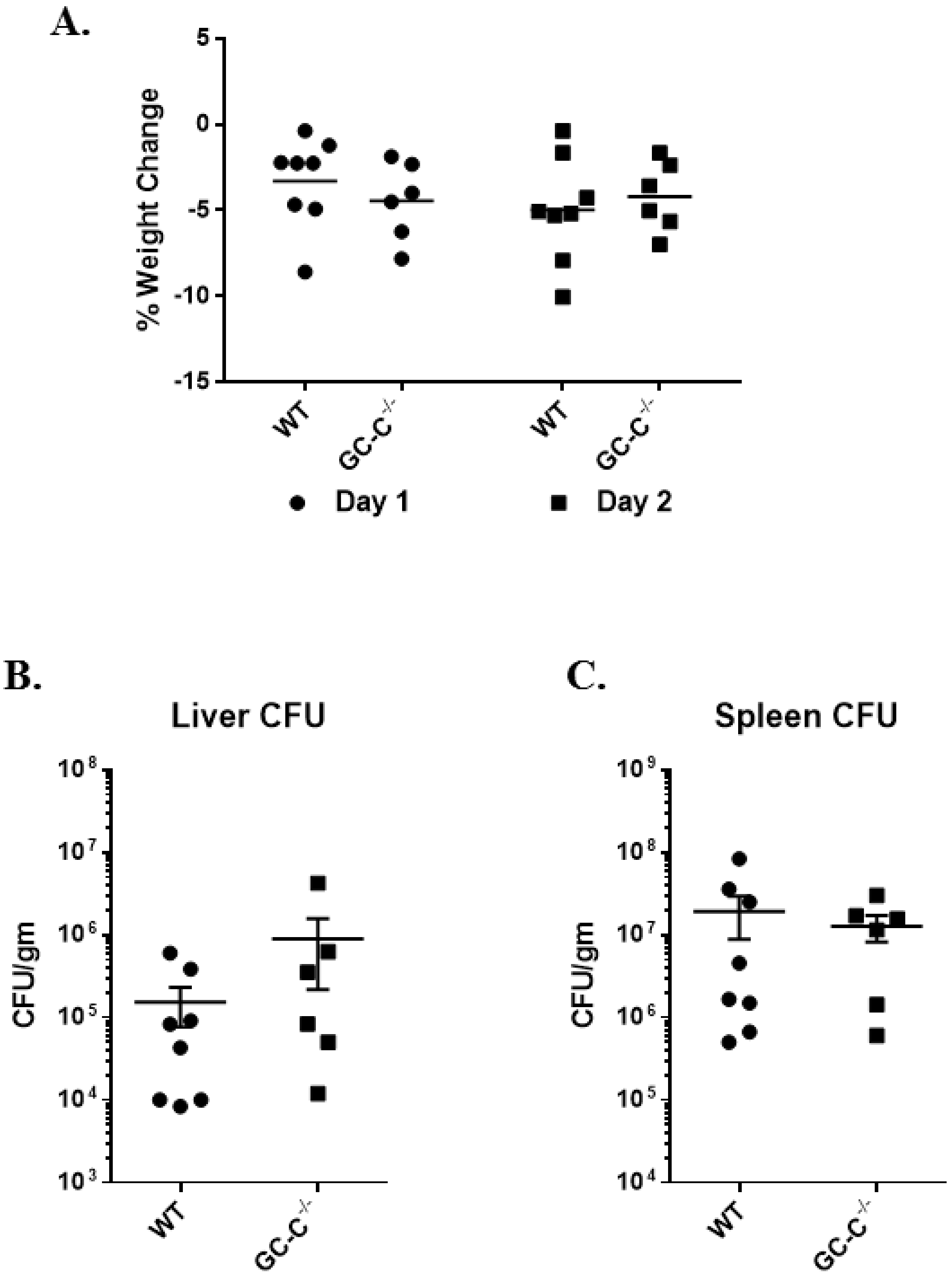

Supplemental Figure S2. GC-C-mediated host defense during *Salmonella* infection. During *Salmonella* infection, wildtype intestine (left side of figure) utilizes GC-C signaling to minimize host-pathogen interaction through well-regulated chloride and bicarbonate secretion that supports a robust mucus layer. Several mechanisms may underlie this role including regulation of protein kinase A and G, control of membrane channels (CFTR, NHE3, CNG (cyclic nucleotide gated)), as well as actin cytoskeleton dynamics via PKG targeting of VASP. In the absence of GC-C (right side of figure), diminished mucus production and/or expansion as well as reduced electrolyte secretion leads to pronounced epithelia-*Salmonella* interactions and increased invasion of the gut by the pathogen.

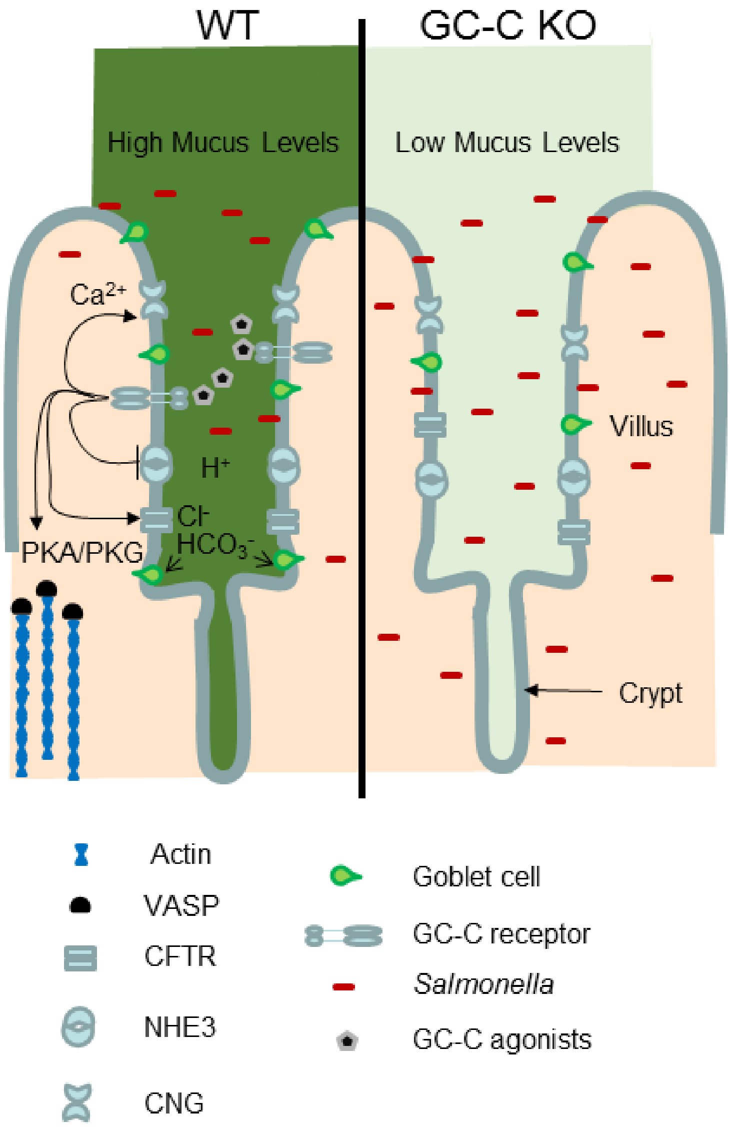

Immunoblot images

Figure 1A: GC-C and TF2B

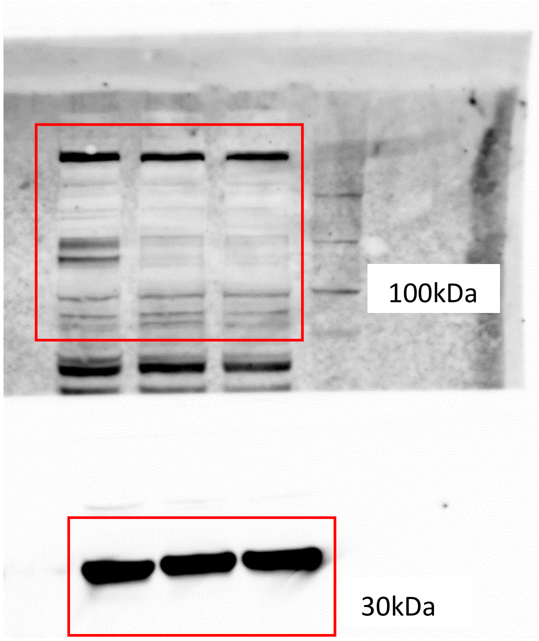

Figure 2C

pVasp (Ser239)

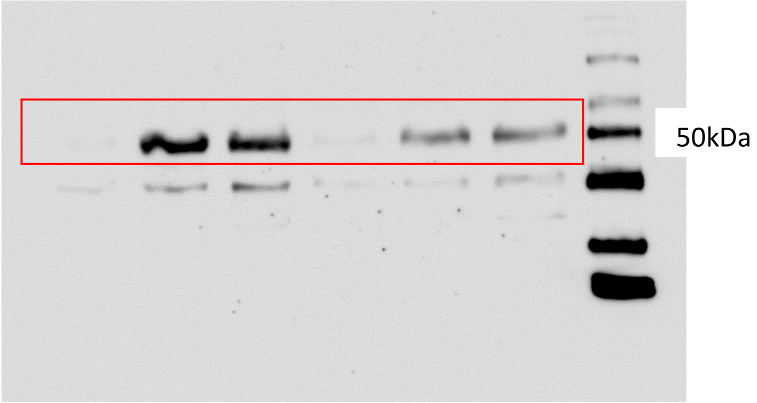

pVasp (Ser157)

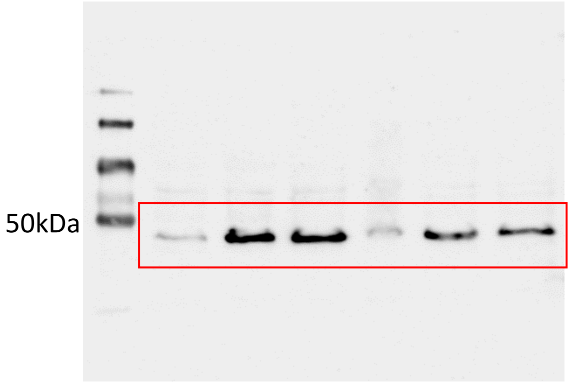

TFIIB

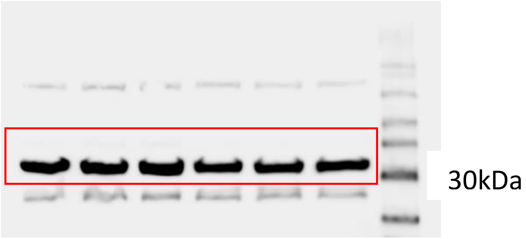

Figure 6D

GC-C

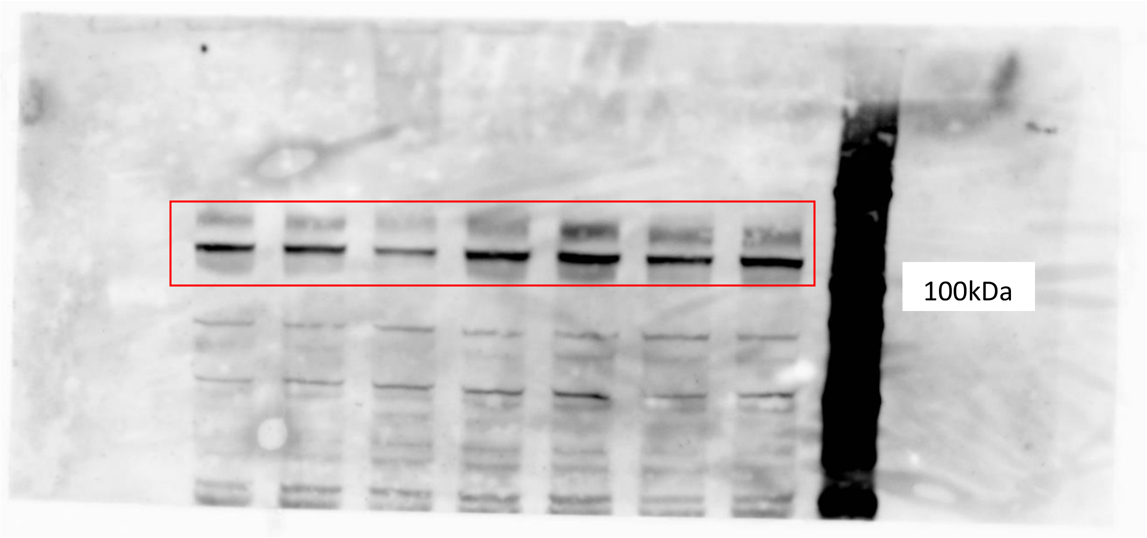

TFIIB

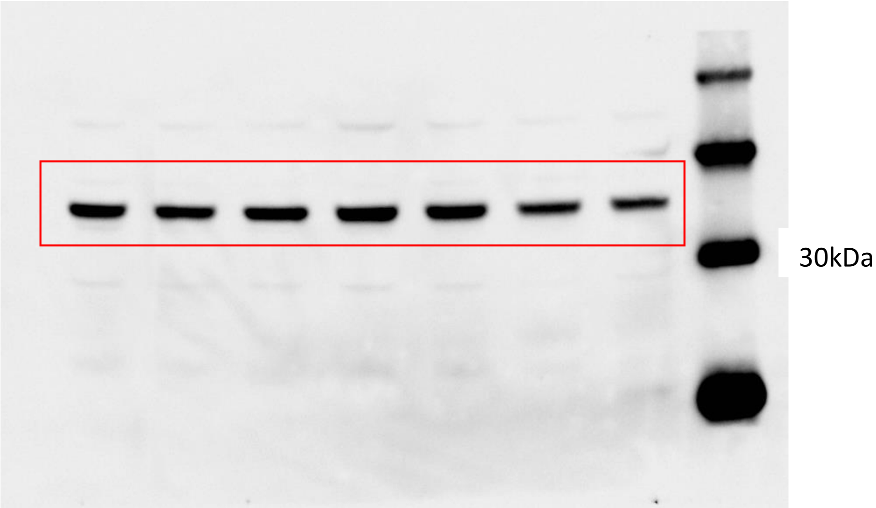

Supplement: Supplementary file 1 — Supplementary Information [file 41598_2018_19868_MOESM1_ESM.pdf]
